# Dr. Edward Constant Seguin

**Published:** 1898-03

**Authors:** 


Dr. Edward Constant Seguin, of New York, died at his home, No. 47 West Fiftieth street, Saturday, February 19, 1898, in the fifty-fifth year of his age. He was born at Paris in 1843, was the only child of Dr. Edward O. Seguin. He graduated from the college of physicians and surgeons in New York in 1864. Meanwhile, he had served in the medical department of the army, first as hospital dresser, then medical cadet, and finally as assistant surgeon U. S. volunteers.

Dr. Seguin became one of the most distinguished specialists in diseases of the nervous system, and has written many scientific papers pertaining to that department of medicine. He was one of the founders of the American Neurological Association and of the New York Neurological Society ; and these, with the New York Pathological Society received most of his attention. He was also a member of several other American and European medical societies.

Dr. Seguin was a man of affable manners, an ornament to the profession, and he will be sadly missed by his professional colleagues and friends.

